# Network-based risk measurements for interbank systems

**DOI:** 10.1371/journal.pone.0200209

**Published:** 2018-07-12

**Authors:** Yongli Li, Guanghe Liu, Paolo Pin

**Affiliations:** 1 School of Business Administration, Northeastern University, Shenyang, P.R.China; 2 Business School, Sun Yat-Sen University, Guangzhou, P.R.China; 3 Department of Decision Sciences, Innocenzo Gasparini Institute for Economic Research, and Bocconi Institute for Data Science and Analytics, University of Bocconi, Milano, Italy; Indiana University, UNITED STATES

## Abstract

This paper focuses on evaluating the systemic risk in interbank networks, proposing a series of measurements: risk distance, risk degree and m-order risk degree. The proposed measurements are formally proven to have good basic and extended properties that are able to reflect the effect of bank size, liability size, liability distribution, and the discount factor on the default risk, not only of a single bank, but also of the entire system. Additionally, the abovementioned properties and the relationship between risk distance and financial contagion indicate the rationality embodied in the proposed measurements. This paper also provides some implications on how to decrease or prevent the systemic risk in an interbank system.

## 1 Introduction

Since the Asian financial crisis of 1997, special attention has been paid to the role of the growing interconnectedness between financial institutions among the many factors that affect financial contagion [1,2]. Particularly after the global crisis of 2007–08, the architecture of financial system building on the abovementioned interconnectedness was viewed as being crucial for its central role in the financial contagion [3–5]. In fact, the abovementioned interconnectedness between financial institutes constitutes the edge of a financial network and the corresponding financial institutes are regarded as the nodes. Particularly, following numerous studies in this field such as [6] and [7], we also focus on the interbank system that can be considered as a fundamental structure for complex financial systems. Note that interbank borrowing and loans, if any, form the abovementioned interconnectedness that link the corresponding banks in the interbank network [8]. The network representation allows to study propagation of failures: recalling the two mentioned financial crisis, for example, one bank’s insolvency may lead to the default cascades in the interbank network. Here, two periods are considered: several banks are assumed to default in the first period and the set of these banks is named initial default set, and then in the second period, some of the remaining banks may be induced to default because of the existing borrowing and loan links. Facing this phenomenon, we want to explore **two problems**. The first one is when one bank’s insolvency occur, which bank will be the next victim? The second one is which initial default set will cause the largest amount of banks to default in the second period? This paper will inherit the idea of risk propagation in networks [9] to cope with the two problems by way of providing a series of convenient measurements.

More concretely, if the answer of the first question is known in advance of financial contagion, we can inject liquidity into the more susceptive banks to avoid the spread of the crisis. As **the first contribution**, we provide a new measurement named risk distance, with the property that a shorter risk distance with the given initial default set means a higher likelihood of default. Among a growing literature on risk analysis in financial networks, the harmonic distance presented by [10] is noteworthy because it captures the susceptibility of each bank to the distress of any other, so that it functions similarly to our proposed risk distance. However, the risk distance that we define is different from the harmonic distance in two aspects: one is that our risk distance considers the cash and marketable assets carried by the banks so that the analysed banks can be heterogeneous, the other is that the risk distance defined here does not assume that the initial default set only contains a single bank. As a result, we prove that our newly proposed risk distance has several different properties with the famous harmonic distance in the following parts of this paper. Overall, our risk distance is a node-level (or say microscopic) indicator that reflects the default likelihood of the remaining banks given an initial default set.

Besides, the second question aims to find the “important” banks from the perspective of financial system risk. To that end, this paper further provides a second new measurement that can reflect the amount of “damage” caused by the initial default set, which is **the second contribution**. Then, the “damages” caused by different initial default sets of the same size can be compared to find which initial default set is most harmful. In particular, when the initial default set contains only one bank, the above problem can be simplified into finding the critical node and measuring its influence on causing financial contagion. Intuitively, the famous Katz-Bonacich centrality can provide the basic idea concerning how to address such a problem [11,12]. With Katz-Bonacich centrality’s becoming conventional wisdom [13,14], we attempt to inherit the basic framework of this centrality and to further develop it to solve the new problem, now that the classical Katz-Bonacich centrality cannot be directly adopted here [15]. The key challenge of solving this problem is how to establish a new matrix that fully reflects the financial system in order to replace the classical adjacent matrix used in Katz-Bonacich centrality when an initial default set is given. Fortunately, [16] has established a Markov transfer matrix with absorbing states, which inspires us on how to obtain a system matrix with the information of the initial default set. As a result, this paper successfully proposes the risk degree of a given initial default set and the risk degrees of an *m*-order initial default set, which reflects the default risk caused by a given initial default set and the maximal system risk caused by all possible default sets with any *m* banks, respectively. Overall, the newly provided measurements, called risk degree and *m*-order risk degree, belong to the type of system-level (i.e., macroscopic) indicators that reflect the collapsing force of the default sets.

Furthermore, apart from the three newly established measurements, this paper also focuses on uncovering these measurements’ properties and demonstrating their rationale, which constitutes **the third contribution**. Specifically, our provided measurements are considered as a function of liability size, liability distribution, bank size and the discount factor, and we further find a much deeper relationship between the provided measurements and the number of failed banks caused by financial contagion. By extending the stylized setup used in papers such as those by [17–19], we also represent interbank systems that consists of *n* banks that are linked via unsecured debt contracts. Finally, abstracting from the background of finance, the nodes of our model have analogies with nodes of information in processes where knowledge is shared instead of risk [20]. So, in the future, managers or government officers monitoring the financial system may be inspired by more general results that fit this analogy.

The rest of this paper is organized as follows, to present the abovementioned ideas and contributions. Section 2 starts from the balance sheet of inter-banks and then defines the three new measurements based on the payment balance of these banks. Section 3 provides the basic properties of the proposed measurements, and Section 4 further proves the extended properties, which are useful to validate the rationality of the proposed measurements. Section 5 concludes and discusses the managerial implications.

## 2 Network-based risk measurements

### 2.1 Balance sheet and payment equilibrium

**Fig 1:**
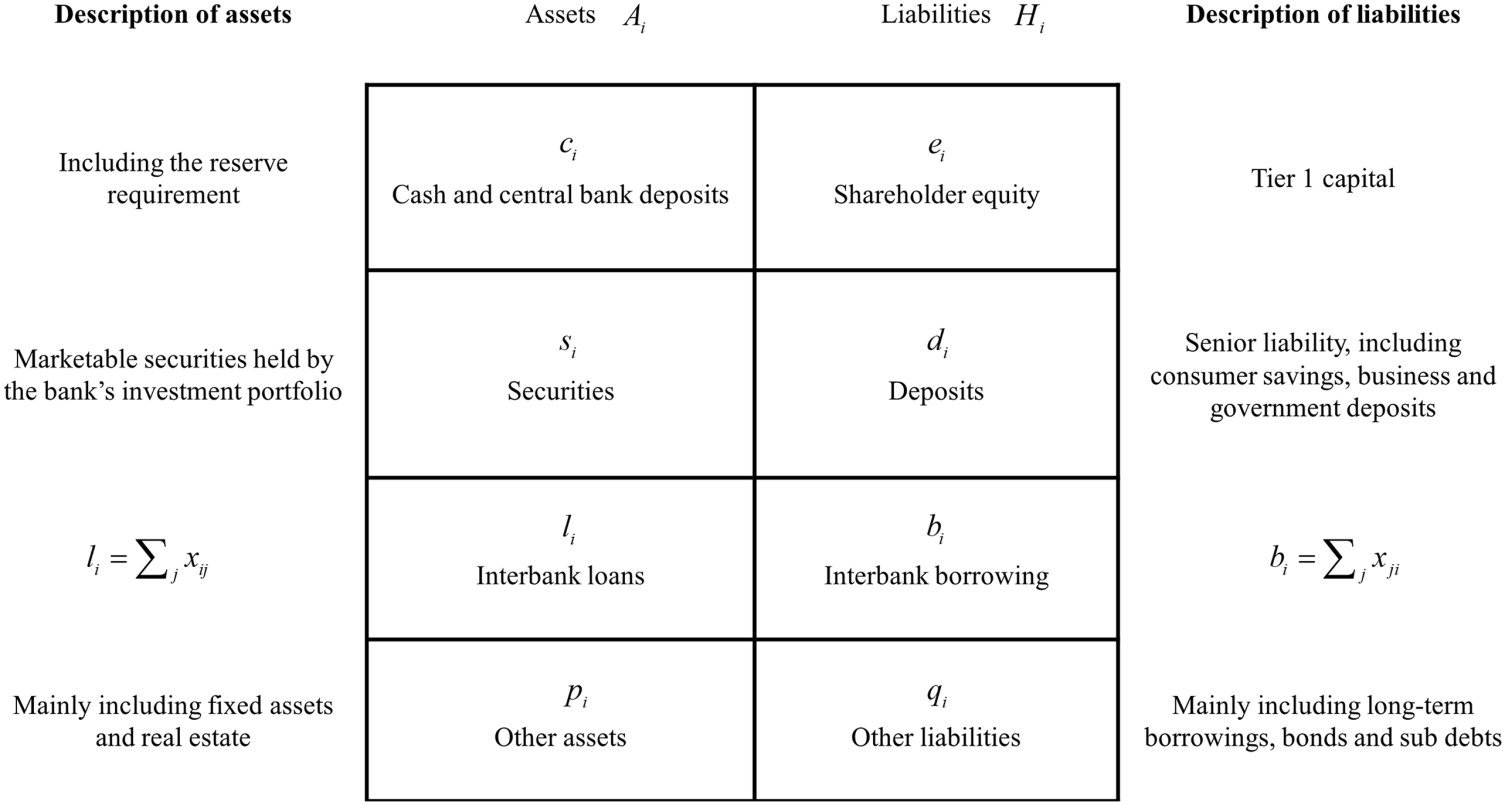
Balance sheet of a stylized commercial bank.

An interbank system is considered here, which consists of several banks that are linked by interbank lending via unsecured debt contracts signed at the initial period. To better clarify the risk contagion process, we start with the balance sheet of a stylized commercial bank within the interbank system. As illustrated in Fig 1, bank *i*’s total assets *A*_*i*_ are composed of liquidities *c*_*i*_, securities *s*_*i*_, interbank loans *l*_*i*_ and other assets *p*_*i*_; thus, the following equation holds:

$$A_{i}=c_{i}+s_{i}+l_{i}+p_{i}.$$ (1)

Meanwhile, bank *i*’s total liabilities *H*_*i*_ consist of shareholder equity *e*_*i*_, deposits *d*_*i*_, interbank borrowing *b*_*i*_ and other liabilities *q*_*i*_:

$$H_{i}=e_{i}+d_{i}+b_{i}+q_{i}.$$ (2)

In fact, the risk in interbank payment systems can originate from different factors; for example, [21] discussed how a shock to deposits could lead to the bank’s default on interbank borrowing or even part of its retail deposits, and [22] considered that the uncertain returns on banks’ securities and other assets were likely to cause a system risk. Unlike the abovementioned studies, this paper does not distinguish the reasons that cause the risk, but rather focuses on the bank’s default induced by any possible initial shocks. Moreover, we further assume that each bank embedded in the interbank system have borrowing or loans at least with the other one bank, namely *b*_*i*_ > 0 and *l*_*i*_ > 0, because the banks without any interbank lending and borrowing are isolated nodes and should not be considered into the interbank system.

Note that the liabilities displayed in the balance sheet can be divided into the senior type and the junior type, and therefore they should be repaid in a different order when shocks occur. Here, the deposits *d*_*i*_ have seniority relative to the bank’s other liabilities; in other words, the available liquidities of bank *i* should repay *d*_*i*_ first and then *b*_*i*_ as well as the other liabilities. Furthermore, the other liabilities generally contain long-term borrowing, bonds and sub debts that really exist as a commercial bank’s liabilities as displayed in Fig 1. Although interbank borrowings and other liabilities mentioned above both belong to the junior liabilities, interbank borrowings are always short-term and other liabilities are often long-term. Thus, we consider that interbank borrowings should be repaid firstly and immediately compared to other liabilities.

Let *al*_*i*_ denote the total available liquidities of bank *i*, and then regardless of the reasons that cause the shocks, two cases may exist: (1) if *al*_*i*_ < *d*_*i*_, bank *i* is called complete default. In this case, the total available liquidities of bank *i* are not enough to pay its senior liabilities; (2) if *d*_*i*_ ≤ *al*_*i*_ < *d*_*i*_ + *b*_*i*_, bank *i* is called part default, which means that, in this case, senior liabilities can be paid in full and the junior creditors are repaid in part. Furthermore, let *x*_*ij*_ (*j* ≠ *i*) denote the amount of money borrowed by bank *j* from bank *i*. When bank *j* is in ***part default***, the amount of money repaid by bank *j* to bank *i* (denoted as *y*_*ij*_) is in proportion to their contract *x*_*ij*_. In mathematics, we have

$$y_{ij}=\frac{x_{ij}}{\sum_{i}x_{ij}}\left(al_{j}-d_{j}\right)=\frac{x_{ij}}{b_{j}}\left(al_{j}-d_{j}\right).$$ (3)

Summarizing the two cases above, *y*_*ij*_ can be further expressed as

$$y_{ij}=\frac{x_{ij}}{b_{j}}\text{max}\left\{0,\text{min}\left\{al_{j}-d_{j},b_{j}\right\}\right\},$$ (4)

where *x*_*ij*_ implies the network structure imbedded in the interbank payments, and therefore their interdependence may induce a cascade of defaults when one or more banks default. Then, Eq (4) will be useful in determining the payment equilibrium because part default actually exists when financial contagion occurs in interbank payments.

Furthermore, because rapid liquation is costly in most cases, bank *i* can only recover a fraction *η*_*i*_ < 1 of the securities *s*_*i*_ and other assets *p*_*i*_, where the defined fraction *η*_*i*_ is defined as the discount factor of bank *i*. According to the expression of repaid money shown in Eq (4), the total available liquidities of bank *i* can be expressed as

$$al_{i}=c_{i}+\sum_{j}^{}y_{ij}+\eta_{i}(s_{i}+p_{i}).$$ (5)

Here, ∑_*j*_*y*_*ij*_ denotes the realised payments made by all the other banks.

To sum up, Eq (4) is a rule that a bank returns the interbank borrowings when facing default, and Eq (5) measures the total available liquidity of a bank. By considering Eqs (4) and (5) together, it seems that the fix-point method can directly be adopted by using the framework of [1], but we do not adopt it in our model setting because the fix-point method ignores the time process of risk contagion to some extent and potentially assumes that all the banks can make the optimal decisions at the same time when the default risk appears in the system. Parallel to these studies related to DebtRank [23,24], this paper also pays attention to the dynamic process of risk contagion and provides the dynamic mechanism as displayed in the next subsection. Besides, this paper defines and validates several risk distances without needing all the banks make the optimal decisions at the same time, and therefore the method of this paper is quite different with fix-point method.

### 2.2 Mechanism of risk contagion

**Fig 2:**
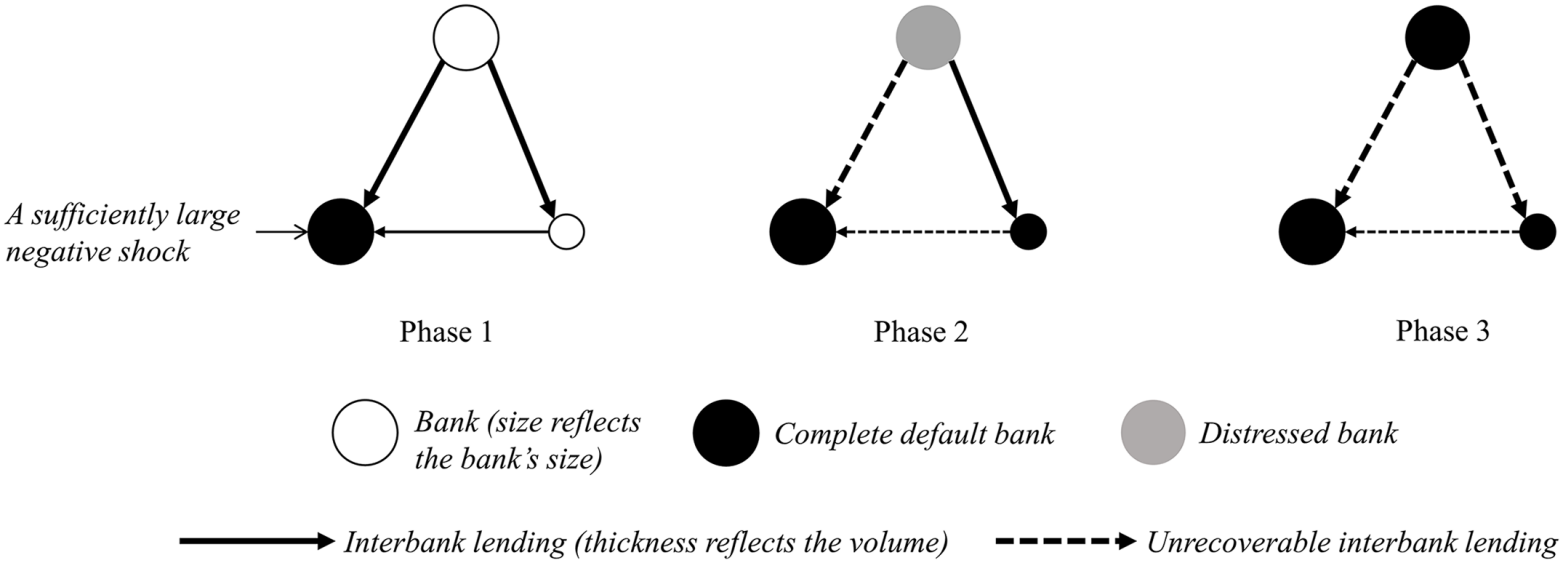
Sketch map of risk contagion.

In order to make clear the mechanism of risk contagion captured by this paper, Fig 2 provides a visual and simple representation, where the interbank system consists of three banks with different bank sizes and these banks are linked by their lending-borrowing relationship. Here, the process of risk contagion can be roughly divided into three successive phases.

As Phase 1 displays, one of the banks suffers a sufficiently large negative shock so as to completely default, and thus the other banks’ loans to this bank become unrecoverable. As a result, the unrecoverable loads cause the bank with bigger size distressed and the one with the smaller size completely default in Phase 2. Subsequently, the complete default of the smaller bank causes the remaining bank’s loan to it unrecoverable so that the remaining bank can also default at this time as shown in Phase 3. Although the real interbank system always contain more banks than our simple example, the mechanism of risk contagion is similar. Note that when part default appears, Eqs (4) and (5) are useful to determine how much repaid money can be received within the mechanism of risk contagion displayed in Fig 2.

### 2.3 Risk distance, risk degree and m-order risk degree

The considered interbank system consists of *n* banks indexed by **N** = {1, 2, ⋯, *n*}, and the lending- borrowing matrix, denoted as **X**, is expressed as **X** = [*x*_*ij*_]_*i*,*j*∈**N**_, where *x*_*ij*_ (*j* ≠ *i*) denote the amount of money borrowed by bank *j* from bank *i* (we pose *x*_*ii*_ = 0). Based on the defined matrix **X**, the deduced system matrix **G** = [*g*_*ij*_]_*i*,*j*∈**N**_ is defined as below.

$$\{\begin{array}{c} g_{ii}=\frac{c_{i}+\eta_{i}(p_{i}+s_{i})}{c_{i}+\eta_{i}(p_{i}+s_{i})+\sum_{k\neq i}x_{ik}}\;\text{when}\;j=i;\\ g_{ij}=\frac{x_{ij}}{c_{i}+\eta_{i}(p_{i}+s_{i})+\sum_{k\neq i}x_{ik}}\;\text{when}\;j\neq i. \end{array}$$ (6)

Here, *g*_*ij*_ (*i* ≠ *j*) represents the ratio of the amount of money lent from bank *i* to bank *j* to bank *i*’s total the total available liquidities. Then, it is not difficult to find that *g*_*ij*_ ≥ 0 (*j* ∈ **N**) and ∑_*j*∈**N**_*g*_*ij*_ = 1 so that the deduced matrix **G** can be understood as a Markov transfer matrix. Note that the above designed matrix does not contain *y*_*ij*_ expressed in Eq (4), but is dependent on *x*_*ij*_, which implies that the designed matrix **G** does not change once the balance sheet is given. In fact, the unchanged **G** facilitates the calculations because we do not need to change **G** frequently with the change of *y*_*ij*_ (*y*_*ij*_ is endogenous variable of our model). Besides, in order to check the designed **G** is good or not, we test the designed **G** whether to satisfy much more desirable properties. In other words, if the designed G can meet much more good properties in measuring the system risk of risk contagion, it is a good design; otherwise, we should try the other forms of **G**. To be honest, the reported **G** in Eq (6) is selected from numerous possible forms and is found to meet much more desirable properties compared to the other tried forms, which will be presented in details in the latter part of this paper.

Let **S** denote the set of initial default banks; for example, if the initial default set only contains bank *j*, then **S** = {*j*}, and if the initial default set contains banks *i* and *j*, then **S** = {*i*, *j*}. The risk distance from a non-default bank to the initial default set **S** is given in Definition 1.

[**Definition 1**] (risk distance). The risk distance from each non-default bank to the initial default set **S** is expressed in the vector **R**_−s_:

$$R_{-S}=\frac{{\left[I-\beta G_{-S}^{}\right]}^{-1}\cdot 1^{\text{T}}}{n},$$ (7)

Where 0 < β < 1 guaranteeing that the inverse matrix exists, **I** is an #(**N** − **S**) × #(**N** − **S**) identity matrix, **1** is an #(**N** − **S**) vector consisting of 1, and **G**_−s_ is **G** deleting the rows and the columns of the banks contained in the set **S**. In addition, the *i*th element of **R**_−s_ is denoted as *r*_*i*,**S**_ that represents the risk distance from *i*th bank to the default bank set **S**.

By recalling the defined Eq (6), the normalization is a useful procedure, whose theoretical basis is Markov chain, by noting that the normalized matrix can be understood or regarded as a Markov transfer matrix. If the status of default is regarded as the absorbing state, the defined risk distance in Eq (7) can be similarly understood as a likelihood that each remaining bank does not reach the absorbing state, according to the principle of finite-state Markov chain. Thus, a longer risk distance means a lower likelihood of default, which accords with our common sense. Interestingly, this form is quite similar to the famous Katz-Bonacich centrality in the field of network science [11].

Further, taking *g*_*ij*_ (*i* ≠ *j*) as an example without loss of generality, the mechanism of risk contagion explained in Section 2.2 means the following statements: when bank *i* face the risk of complete default, bank *j* should first returned the money borrowed from bank *i*, so *g*_*ij*_ = 0 at this time; and then even if bank *i* has received all the money that was lent out to the other banks, bank *i* can also completely default so that it appears in the initial default set **S**, and at this time, *g*_*ij*_ = 0 because complete default means that bank *i* cannot return the money borrowed from bank *j* (∀*j* ≠ *i*). By considering the above mechanism, we should delete the rows and the columns of the banks in the initial default set **S**, which interprets why we adopt the **G**_−s_ to calculate the risk distance in Eq (7).

As a result, the normalization is important here for three reasons. First, the normalization guarantees that the sum of elements in each row equals 1, which accords with the definition of Markov transfer matrix, and therefore, the principle of Markov transfer matrix can be adopted directly as we have explained. Second, the normalization makes each element in the normalized matrix reflect the ratio of the corresponding liabilities, so that the bank size is potentially considered. In fact, the bank size, defined as the total amount of assets (or liabilities) of one bank, is an important factor influencing the risk contagion. To make it clear, if two banks borrow the same amount of money from another bank but the two banks have different sizes, the normalization will shows different ratios of the borrowed money in the two banks, but without the normalization, the effect of bank size cannot be reflected. Moreover, the normalization can also reflect the effect from each bank’s discount factor *η*_*i*_ (∀*i* ∈ **N**), which is also important in the process of risk contagion.

Subsequently, let *rv*(**S**) denote the risk degree of the whole interbank system given the initial default set **S**. Based on the defined risk distance (see Definition 1), *rv*(**S**) is defined as follows.

[**Definition 2**] (risk degree). Given the initial default set **S**, the risk degree *rv*_**s**_ is defined as

$$rv_{S}={\left(1\cdot R_{-S}\right)}^{-1},$$ (8)

where **1** is an #(**N** − **S**) vector whose elements are all 1. In other words,**1** ⋅ **R**_−s_ equals the sum of all of the elements in **R**_−s_

Recalling the explanations of Definition 1, the longer risk distance means a lower likelihood of default. Here, we first sum all the distances from the remaining banks to the given initial default set, and accordingly the mathematical expression is **1** ⋅ **R**_−s_. Note that a larger value of **1** ⋅ **R**_−s_ means a lower system risk degree, which does not accord with our common sense. Thus, we use (**1** ⋅ **R**_−s_)^−1^ to measure the system risk degree to avoid this problem.

Furthermore, from the perspective of the system, we can compare the risk degrees relative to all possible initial default sets and determine the maximal risk degree and the corresponding default set. Considering the amount of default banks that will affect the risk degree, we keep this amount identical for fair comparison. As a result, let *rv*_*m*_(**G**) denote the *m*-order risk degree of the given system **G**, where *m* is the number of banks contained in the given initial default set **S**. For example, when *m* = 2, *rv*_2_(**G**) means the maximum sum of risk distance from each non-default bank to any possible default bank sets which contain two banks. Then, the *rv*_*m*_(**G**) is defined as below, where *m* = 1, 2, ⋯, *n*.

[**Definition 3**] (m-order risk degree). For all initial default sets containing *m* banks, the maximal risk degree among all initial default sets is defined as the *m*-order risk degree, whose mathematical expression is

$$rv_{m}(G)=\underset{i_{1},i_{2},\cdots ,i_{m}\in N}{\text{max}}{\left(1\cdot R_{-\{i_{1},i_{2},\cdots ,i_{m}\}}\right)}^{-1},$$ (9)

Where *i*_1_, *i*_1_, ⋯, *i*_*m*_ are the different banks contained in the initial default set and, therefore, the total number of their combination is *n*!/(*m*!(*n* − *m*)!).

Keeping the number of banks in initial default set identical, the defined *m*-order risk degree identifies not only the maximal risk degree for all possible initial default sets containing *m* banks but also the corresponding default set that leads to the highest system risk.

## 3 Basic properties

This section aims to provide the basic properties of the proposed three measurements. In detail, these basic properties include the non-negativity, the asymmetry and the monotonicity of the defined risk distance, the monotonicity of the risk degree, and the heterogeneity of different-order risk degrees. Although these basic properties seem natural at first glance, they can not only deepen our understanding of the proposed measurements but also illustrate the rationality of these measurements.

[**Property B1**] (Non-negativity of risk distance). For any given system **G** and the initial default set **S**, the risk distance defined in Eq (7) and the corresponding risk degree defined in Eq (8) are both non-negative.

**Proof**. Because each element of **G**_−**S**_ ⋅ **1**^T^ is no more than 1 and 0<*β*<1, it holds that

$${\text{[}I-\beta G_{-S}]}^{-1}=I+\beta G_{-S}+\beta^{2}G_{-S}{}^{2}+\cdots .$$ (10)

Eq (10) clearly shows that all of the elements in [**I** − *β***G**_−**S**_]^−1^ are non-negative since all of the elements in **G**_−s_ are non-negative. Thus, Property B1 is immediately obtained.

[**Property B2**] (Asymmetry of risk distance). The risk distance from the bank *i* to the initial default set {*j*} must not be equal to that from bank *j* to the initial default set {*i*}.

**Proof**. Here, we provide an example to show that *r*_*i*,{*j*}_ ≠ *r*_*j*,{*i*}_. Given *β* = 0.9, the corresponding system matrix of the three banks is set as below:

$$\left(\begin{array}{ccc} 0.5 & 0.3 & 0.2\\ 0.1 & 0.3 & 0.6\\ 0.4 & 0.4 & 0.2 \end{array}\right).$$

As a result, *r*_2,{1}_ = 3.36 and *r*_1,{2}_ = 2.59, which immediately obtains the result.

[**Property B3**] (Monotonicity of risk distance). For any given system **G**, it holds that *r*_*i*,{*s*}_ ≥ *r*_*i*,{*s*,*t*}_, where *i* ≠ *s* and *t* ≠ *i*, *s*.

**Proof**. According to Eqs (7) and (10), we have

$$r_{i,\{s\}}-r_{i,\{s,t\}}=\frac{\beta g_{it}+\beta^{2}\left[\sum_{l\neq s,t}g_{il}g_{lt}+\sum_{k\neq s}g_{ki}g_{it}\right]+\cdots }{n}.$$ (11)

If only considering the path starting from *t* and ending at *i*, we can further have

$$r_{i,\{s\}}-r_{i,\{s,t\}}\geq \frac{\sum_{l=1}^{+\infty }\beta^{i}g_{it}^{\left[l\right]}}{n},$$ (12)

where $g_{it}^{\left[l\right]}$ means the product of all of the elements in the path starting from *i* and ending at *t* with the length of *l* in **G**_−s_. Then, because *g*_*ij*_ (*i*, *j* ∈ **N**) are all non-negative, it is not difficult to determine that *r*_*i*,{*s*}_ ≥ *r*_*i*,{*s*,*t*}_.

The above three basic properties of the defined risk distance show that (1) the defined risk distance is non-negative, which accords with our common sense; (2) banks takes different effect on the system risk or financial contagion according to the different positions in the networks and thus have an asymmetrical risk distance; and (3) adding one more bank to the initial default set will not increase the risk distance, which is true because much greater bank defaulting in the first period often implies much greater fragility for the entire system.

[**Property B4**] (Monotonicity of risk degree). For any given financial system **G**, it holds that *rv*_*m*+1_(**G**) ≥ *rv*_*m*_(**G**), where *m* = 1, 2, ⋯, *n*.

**Proof**. For any {*i*_1_, *i*_2_, ⋯, *i*_*m*_} and {*i*_1_, *i*_2_, ⋯, *i*_*m*_}\{*i*_*k*_}(*k* = 1, 2, ⋯, *m*), it holds that

$$1\cdot R_{-\{i_{1},i_{2},\cdots ,i_{m}\}}\leq 1\cdot R_{-\{i_{1},i_{2},\cdots ,i_{m}\}\backslash \{i_{k}\}},$$ (13a)

because of the proven monotonicity of risk distance in Property B3. Then, we have

$${\left(1\cdot R_{-\{i_{1},i_{2},\cdots ,i_{m}\}}\right)}^{-1}\geq {\left(1\cdot R_{-\{i_{1},i_{2},\cdots ,i_{m}\}\backslash \{i_{k}\}}\right)}^{-1},$$ (13b)

because their maximum values inherit the inequality relation. Then, Inequality (13b) guarantees that *rv*_*m*+1_(**G**) ≥ *rv*_*m*_(**G**).

[**Property B5**] (Heterogeneity of different-order risk degrees). For two given financial systems **G**_1_ and **G**_2_ whose node number is *n*_1_ and *n*_2_, respectively, if *rv*_1_(**G**_1_) ≤ *rv*_1_(**G**_2_), then it must not hold that *rv*_*m*_(**G**_1_) ≤ *rv*_*m*_(**G**_2_), where 2 ≤ *m* ≤ min{*n*_1_, *n*_2_}.

**Proof**. Similar to the proof of Property B2, we here provide an example to illustrate that *rv*_1_(**G**_1_) < *rv*_1_(**G**_2_) and *rv*_2_(**G**_1_) > *rv*_2_(**G**_2_) can exist simultaneously. Additionally, given *β* = 0.9, the two system matrices are shown below. According to Definition 3, we have *rv*_1_(**G**_1_) = 0.0286, *rv*_1_(**G**_2_) = 0.0342, *rv*_2_(**G**_1_) = 0.10, and *rv*_2_(**G**_2_) = 0.0950, meaning that *rv*_1_(**G**_1_) < *rv*_1_(**G**_2_) and *rv*_2_(**G**_1_) > *rv*_2_(**G**_2_) can exist simultaneously. Thus, Property B5 holds.

$$G_{1}=\left(\begin{array}{cccccc} 0.5 & 0.5 & & & & \\ 0.5 & 0.5 & & & & \\ & & 0.25 & 0.25 & 0.25 & 0.25\\ & & 0.25 & 0.25 & 0.25 & 0.25\\ & & 0.25 & 0.25 & 0.25 & 0.25\\ & & 0.25 & 0.25 & 0.25 & 0.25 \end{array}\right),\;\text{and}\;G_{\text{2}}=\left(\begin{array}{cccccc} 1/3 & 1/3 & 1/3 & & & \\ 1/3 & 1/3 & 1/3 & & & \\ 1/3 & 1/3 & 1/3 & & & \\ & & & 1/3 & 1/3 & 1/3\\ & & & 1/3 & 1/3 & 1/3\\ & & & 1/3 & 1/3 & 1/3 \end{array}\right).$$

Properties B4 and B5 focus on the defined risk degree and m-order risk degree, respectively. Here, Property B4 implies that adding one more bank to the initial default set **S** will not decrease the risk degree of the financial system, and Property B5 indicates that if one system’s 1-order risk degree is larger than another system’s 1-order risk degree, we can’t take for that the system’s higher order risk degree is still larger. In fact, two conflicting perspectives exist in the existing literature: one perspective supports that a more connected network structure enhances the system’s resilience [25], whereas the other suggests that dense interconnections will cause a destabilizing force to decrease the system’s risk [26]. The illustrated Property B5 can explain the conflicting findings from the defined different order risk degree.

## 4 Extended properties

Almost every central bank needs deposit reserves and limits the reserve requirement, i.e. *c*_*i*_ / *d*_*i*_, according to the specific law of its nation. Although the deposit reserve ratio of each bank is slightly different, we can assume a constant ratio of *d*_*i*_ to *H*_*i*_ since empirical analysis finds that such a ratio is almost the same for different banks within one nation [27]. Thus, we make the following assumption.

[**Assumption 1**] For any given system **G**, we assume that *d*_*i*_ = *γH*_*i*_ for all *i* ∈ **N**, where 0 < *γ* < 1.

Based on Assumption 1, for any given initial default set **S**, if the default set **S** leads to the complete default of bank *i* (*i* ∉ **S**) with the risk contagion, then the following inequality will hold:

$$\frac{c_{i}+\eta_{i}(p_{i}+s_{i})+\sum_{j}y_{ij}}{c_{i}+p_{i}+s_{i}+\sum_{j}x_{ij}}<\gamma ,$$ (14)

Where *al*_*i*_ has been defined in Eq (5), meaning that the numerator is the available asset after the risk contagion caused by the initial default set **S** and the denominator is the initial available asset. Furthermore, we next provide several extended properties to demonstrate the rationality of the proposed measurements; in other words, to check whether the proposed measurements can correctly reflect the system risk as the function of liability size, liability distribution, bank size and the discount factor. Additionally, the relationship between financial contagion and risk distance is also explored, which is significant and meaningful for validating the rationality of the proposed measurements.

### 4.1 Relationship between financial contagion and risk distance

First, we explore whether the defined risk distance can reflect the risk level of banks by controlling the effect of the discount factor. To this end, this subsection sets *η*_*i*_ = 1 (*i* ∈ **N**) to focus on the relationship between financial contagion and risk distance. Specifically, given an initial default set **S**, if bank *i* defaults with the financial contagion, then do all the banks that are shorter to the set **S** than bank *i*? The following Property E1 gives the answer.

[**Property E1**] For any given initial default set **S** and two banks *i* and *j* (*i*, *j* ∉ **S**) in a given system **G** with *η*_*i*_ = 1 for any *i* ∈ **N**, if bank *j* completely defaults with the financial contagion and *r*_*i*,**S**_ < *r*_*j*,**S**_ for any *β* ∈ (0, 1), then bank *i* must completely default. In addition, there exists Δ*r*, such that bank *w* defaults if *r*_*w*,**S**_ < Δ*r*.

**Proof**. For any given initial default set **S** and *η*_*i*_ = 1 for any *i* ∈ **N**, Eq (5) and Assumption 1 guarantee that the necessary and sufficient condition of bank *j* completely defaulting is

$$al_{j}\sum_{k\notin S}g_{jk}<\gamma al_{j}\;\text{or}\;\sum_{k\notin S}g_{jk}<\gamma ,$$ (15)

Recalling Eqs (3) and (5), Inequality (15) equals

$$1_{j}(I-G_{-S}^{})1_{}^{\text{T}}>1-\gamma .$$ (16)

On the other hand, *r*_*i*,**S**_ < *r*_*j*,**S**_ can be further expressed as

$$(1_{i}-1_{j})\cdot {\left[I-\beta G_{-S}^{}\right]}^{-1}\cdot 1_{}^{\text{T}}<0,$$ (17)

and the Taylor expansion guarantees that

$$(1_{i}-1_{j})\cdot (G_{-S}^{}+\beta G_{-S}^{2}{\left[I-\beta G_{-S}^{}\right]}^{-1})\cdot 1_{}^{\text{T}}<0,$$ (18)

which holds for any *β* ∈ (0, 1). Then, let *β* → 0, and we immediately obtain that

$$(1_{i}-1_{j})\cdot G_{-S}^{}\cdot 1_{}^{\text{T}}<0,$$ (19)

which equals **1**_*i*_(**I** − **G**_−**S**_)**1**^T^ > **1**_*j*_(**I** − **G**_−**S**_)**1**^T^ > 1 − *γ*. Thus, the first part of Property E2 holds.

As a deduction, let Δ*r* = max(*r*_*k*,**S**_ | bank *k* defaults), and then the second part of Property E2 is obtained based on the result of the first part.

Property E1 provides the answer of the question is YES. Again, given an initial default set **S**, if bank *i* defaults with the financial contagion, all the banks shorter to the set **S** than bank *i* will default. Thus, the defined risk distance is demonstrated to be reasonable because it can fully reflect the insolvency contagion of banks when banks can liquate their assets freely. Note that the effect of the discount factor will be discussed in Subsection 4.5 to enrich our illustrations.

### 4.2 Risk measurements as a function of liability size

In this subsection, we first check whether the increase in all pairwise liabilities raises the system risk, and the following Lemma 1 gives the answer.

[**Lemma 1**] For any given financial system **G** and an initial default set **S**, let ${\tilde{x}}_{ij}=\alpha x_{ij}$ for all *i* ≠ *j* and *α* > 1 in the new financial system denoted as $\tilde{G}$. If bank *i* (*i* ∉ **S**) completely defaults with the risk contagion in **G**, then it must completely default in $\tilde{G}$.

**Proof**. Here, we first investigate the relationship of the available assets of bank *i* (*i* ∉ **S**) between **G** and $\tilde{G}$, and its results are as follows:

$$\frac{c_{i}+\eta_{i}(p_{i}+s_{i})+\sum_{j}{\tilde{y}}_{ij}}{c_{i}+p_{i}+s_{i}+\sum_{j}{\tilde{x}}_{ij}}\leq \frac{c_{i}+\eta_{i}(p_{i}+s_{i})+\alpha \sum_{j}y_{ij}}{c_{i}+p_{i}+s_{i}+\alpha \sum_{j}x_{ij}},$$ (20)

because of Eq (5) and the fact that $\tilde{al_{i}}<\alpha \cdot al_{i}$. Then, because *α* > 1 and ∑_*j*_*x*_*ij*_ > ∑_*j*_*y*_*ij*_, it is not difficult to obtain that

$$\frac{c_{i}+\eta_{i}(p_{i}+s_{i})+\alpha \sum_{j}y_{ij}}{c_{i}+p_{i}+s_{i}+\alpha \sum_{j}x_{ij}}<\frac{c_{i}+\eta_{i}(p_{i}+s_{i})+\sum_{j}y_{ij}}{c_{i}+p_{i}+s_{i}+\sum_{j}x_{ij}}.$$ (21)

Then, according to Inequality (14), for the given default set **S** and bank *i* (*i* ∉ **S**), it holds that

$$\frac{c_{i}+\eta_{i}(p_{i}+s_{i})+\sum_{j}y_{ij}}{c_{i}+p_{i}+s_{i}+\sum_{j}x_{ij}}<\gamma ,$$ (22)

and Inequalities (20) and (21) guarantee that

$$\frac{c_{i}+\eta_{i}(p_{i}+s_{i})+\sum_{j}{\tilde{y}}_{ij}}{c_{i}+p_{i}+s_{i}+\sum_{j}{\tilde{x}}_{ij}}<\gamma .$$ (23)

Overall, Lemma 1 holds.

The proven Lemma 1 gives us a hint that increasing all pairwise liabilities in the network will raise the systemic risk, because the banks that completely default in **G** also completely default in $\tilde{G}$. Note that, here, we use the number of banks that completely default to depict the level of system risk. Then, we further check whether the defined risk measurements can correctly reflect the change in the system risk when all pairwise liabilities rise, as discussed above. As a result, by keeping all liquidities *c*_*i*_, securities *s*_*i*_ and other assets *p*_*i*_ unchanged, the following Property E2 gives the answer.

**[Property E2]** For any given financial system **G**, if ${\tilde{x}}_{ij}=\alpha x_{ij}$ for all *i* ≠ *j* and *α* > 1 in the new financial system denoted as $\tilde{G}$, for any given initial default set **S**, the defined risk distance is shorter, and the defined risk degree and the m-order risk degree are larger in the new financial system.

**Proof**. Based on the definition of **G** = [*g*_*ij*_]_*i*,*j*∈**N**_ in Eq (5), the relationship between $\tilde{G}$ and **G** is

$$I-{\tilde{G}}_{-S}=T(\alpha )\left[I-G_{-S}\right],$$ (24)

and

$$T(\alpha )=diag\left(\frac{\alpha }{(1-\alpha )g_{11}+\alpha },\frac{\alpha }{(1-\alpha )g_{22}+\alpha },\cdots ,\frac{\alpha }{(1-\alpha )g_{nn}+\alpha }\right).$$ (25)

whose elements are all more than 1. Then,

$$I-\beta {\tilde{G}}_{-S}=T(\alpha )\left[I-\beta G_{-S}\right]+(1-\beta )\left[I-T(\alpha )\right].$$ (26)

Now, $I-\beta {\tilde{G}}_{-S}$ is considered the function of *α*, and we further have

$$\frac{\partial {\left[I-\beta {\tilde{G}}_{-S}\right]}^{-1}}{\partial \alpha }\cdot 1=-{\left[I-\beta {\tilde{G}}_{-S}\right]}^{-1}\frac{\partial \left[I-\beta {\tilde{G}}_{-S}\right]}{\partial \alpha }{\left[I-\beta {\tilde{G}}_{-S}\right]}^{-1}\cdot 1.$$ (27)

On one hand, all of the elements in ${\left[I-\beta {\tilde{G}}_{-S}\right]}^{-1}$ are non-negative, which has been proven in Property B1. On the other hand,

$$\begin{array}{ll} \frac{\partial \left[I-\beta {\tilde{G}}_{-S}\right]}{\partial \alpha } & =\frac{\partial T(\alpha )}{\partial \alpha }\left[I-\beta G_{-S}\right]-(1-\beta )\frac{\partial T(\alpha )}{\partial \alpha }\\ & =\frac{\partial T(\alpha )}{\partial \alpha }{\left[T(\alpha )\right]}^{-1}\left(\left[I-\beta {\tilde{G}}_{-S}\right]-(1-\beta )\left(I-T(\alpha )\right)\right)-(1-\beta )\frac{\partial T(\alpha )}{\partial \alpha }, \end{array}$$ (28)

and therefore, Eq (27) is equivalent to

$$\begin{array}{ll} \frac{\partial {\left[I-\beta {\tilde{G}}_{-S}\right]}^{-1}}{\partial \alpha }\cdot 1 & =-{\left[I-\beta {\tilde{G}}_{-S}\right]}^{-1}\frac{\partial T(\alpha )}{\partial \alpha }{\left[T(\alpha )\right]}^{-1}\cdot 1+(1-\beta ){\left[I-\beta {\tilde{G}}_{-S}\right]}^{-1}\frac{\partial T(\alpha )}{\partial \alpha }{\left[T(\alpha )\right]}^{-1}{\left[I-\beta {\tilde{G}}_{-S}\right]}^{-1}\\ & ={\left[I-\beta {\tilde{G}}_{-S}\right]}^{-1}\frac{\partial T(\alpha )}{\partial \alpha }\left((1-\beta ){\left[I-\beta {\tilde{G}}_{-S}\right]}^{-1}-I\right)\cdot 1. \end{array}$$ (29)

Note that $(1-\beta ){\left[I-\beta {\tilde{G}}_{-S}\right]}^{-1}-I=\beta {\left[I-\beta {\tilde{G}}_{-S}\right]}^{-1}\left({\tilde{G}}_{-S}-I\right)$ and because all of the elements in $\left({\tilde{G}}_{-S}-I\right)\cdot 1$ are less than 0. Thus, all of the elements in ${\left[I-\beta {\tilde{G}}_{-S}\right]}^{-1}\cdot 1$ are decreasing functions of *α*, meaning that the defined risk distance obtains its maximal value when *α* → 1 and obtains its minimum value when *α* → +∞. Furthermore, because the inequality of risk degrees holds for any initial default set **S**, the inequality is naturally inherited by the defined *m*-order risk degree. Thus, Property E2 holds, and we also obtain the boundary of the defined measurements when the liability size changes.

Overall, Property E2 reflects that the rise in pairwise liabilities will increase the systemic risk, regardless of the type of network structure. Hence, controlling the level of total credits in the system is a method of managing systemic risk. From another perspective, the proposed measurements can properly reflect the increase in systemic risk, which indicates that the definition of the proposed measurement is rational.

### 4.3 Risk measurements as a function of liability distribution

Intuitively, a bank with positive net interbank lending is more likely to be harmed by the risk contagion because it will get loss from its counterparties’ default. Accordingly, this subsection focuses on the effect of liability distributions and explores how to arrange the liability distribution among banks to obtain a smaller system risk degree. To this end, consider any given system **G**, and suppose that each bank’s liability, on one hand, is kept unchanged, (that is, keeping ∑_*i* ≠ *j*_*x*_*ij*_ unchanged for any *i* ∈ **N** to avoid the effect from the amount of liability that has been proven influential), and, on the other hand, each bank’s total lending amount is kept equal to its borrowing amount (that is, ∑_*i*_*x*_*ij*_ = ∑_*j*_*x*_*ij*_ to ignore the effect of the difference between the loan and borrowing amounts). This subsection focuses on the effect of liability distributions and explores how to arrange the liability distribution among banks to obtain a smaller system risk degree. The following Property E3 gives the answer.

[**Property E3**] Given any system G with the precondition that ∑_*i*_*x*_*ij*_ = ∑_*j*_*x*_*ij*_ for any *i*, *j* ∈ **N**, let ${\tilde{x}}_{ij}=0.5(x_{ij}+x_{ji})$ for any *i*, *j* ∈ **N** in the newly generated system $\tilde{G}$ so the operation does not change each bank’s liability. Then, if the same default set **S** is given for the two systems, we have $rv_{S}(\tilde{G})\leq rv_{S}(G)$ and the equation holds if and only if *x*_*ij*_ = *x*_*ji*_ holds in the given system **G**.

**Proof**. Here, system $\tilde{G}$ changes the liability distribution of the given system **G** and does not change each bank’s liability, noting that the precondition guarantees the following equation:

$$\sum_{j\neq i}{\tilde{x}}_{ij}=0.5\sum_{j\neq i}(x_{ij}+x_{ji})=\sum_{j\neq i}x_{ij}.$$ (30)

Thus, the above operation, which changes the liability distribution, does not change each bank’s liability. In addition, the above operation does not change *c*_*i*_ + *η*_*i*_ ⋅ (*p*_*i*_ + *s*_*i*_) for any *i* ∈ **N**, meaning that ${\tilde{g}}_{ii}=g_{ii}$. Moreover, we have

$${\tilde{g}}_{ij}=\frac{{\tilde{x}}_{ij}}{\sum_{j\neq i}{\tilde{x}}_{ij}}=\frac{0.5(x_{ij}+x_{ji})}{\sum_{j\neq i}x_{ij}}=0.5(g_{ij}+g_{ji}).$$ (31)

Accordingly, it holds that $\tilde{G}=0.5(G+G^{\text{T}})$.

Knowing *rv*_**S**_(**G**) = (**1** ⋅ **R**_−**S**_(**G**))^−1^, we next focus on the item **1** ⋅ **R**_−**S**_(**G**) whose detailed form is **1** ⋅ (**I** − *β***G**_−**S**_)^−1^ ⋅ **1**^T^ / *n*. Accordingly, the problem of proving $rv_{S}(\tilde{G})\leq rv_{S}(G)$ is changed into the problem of proving $1\cdot {\left[I-\beta {\tilde{G}}_{-S}^{}\right]}^{-1}\cdot 1^{\text{T}}\geq 1\cdot {\left[I-\beta G_{-S}^{}\right]}^{-1}\cdot 1^{\text{T}}$, where ${\tilde{G}}_{-S}^{}=0.5(G_{-S}^{}+G_{-S}^{\text{T}})$. The following part is to prove the above inequality. First, we have

$$\begin{array}{ll} \frac{1_{-S}\cdot {\left[I-\beta G_{-S}\right]}^{-1}\cdot 1_{-S}^{\text{T}}}{1_{-S}\cdot {\left[I-\beta {\tilde{G}}_{{}_{-S}}\right]}^{-1}\cdot 1_{-S}^{\text{T}}} & =\frac{1_{-S}\cdot 0.5({\left[I-\beta G_{-S}\right]}^{-1}+{\left[I-\beta G_{-S}\right]}^{-\text{T}})\cdot 1_{-S}^{\text{T}}}{1_{-S}\cdot {\left[I-\beta {\tilde{G}}_{{}_{-S}}\right]}^{-1}\cdot 1_{-S}^{\text{T}}}\\ & \leq \underset{\|y\|=1}{\text{max}}\frac{y\cdot 0.5({\left[I-\beta G_{-S}\right]}^{-1}+{\left[I-\beta G_{-S}\right]}^{-\text{T}})\cdot y^{\text{T}}}{y\cdot {\left[I-\beta {\tilde{G}}_{{}_{-S}}\right]}^{-1}\cdot y^{\text{T}}}. \end{array}$$ (32)

Then, because 0.5([**I** − *β***G**_−**S**_]^−1^ + [**I** − *β***G**_−**S**_]^−T^) and ${\left[I-\beta {\tilde{G}}_{-S}^{}\right]}^{-1}$ are both symmetric matrices, the theorem of Rayleigh-Ritz guarantees that

$$\begin{array}{c} \underset{\left\|y\right\|=1}{\text{max}}\frac{y\cdot 0.5({\left[I-\beta G_{-S}^{}\right]}^{-1}+{\left[I-\beta G_{-S}^{}\right]}^{-\text{T}})\cdot y_{}^{\text{T}}}{y\cdot {\left[I-\beta {\tilde{G}}_{-S}^{}\right]}^{-1}\cdot y_{}^{\text{T}}}\\ =\lambda_{\text{max}}\left[0.5({\left[I-\beta G_{-S}^{}\right]}^{-1}+{\left[I-\beta G_{-S}^{}\right]}^{-\text{T}})\cdot [I-\beta {\tilde{G}}_{-S}^{}]\right]\\ =\lambda_{\text{max}}\left[0.5({\left[I-\beta G_{-S}^{}\right]}^{-1}+{\left[I-\beta G_{-S}^{}\right]}^{-\text{T}})\cdot 0.5([I-\beta G_{-S}^{}]+{\left[I-\beta G_{-S}^{}\right]}^{\text{T}})\right]\\ =\lambda_{\text{max}}\left[0.5+0.25({\left[I-\beta G_{-S}\right]}^{-1}{\left[I-\beta G_{-S}\right]}^{\text{T}}+{\left[I-\beta G_{-S}\right]}^{-\text{T}}[I-\beta G_{-S}])\right]\\ \leq 0.5+0.25\cdot \lambda_{\text{max}}\left({\left[I-\beta G_{-S}\right]}^{-1}{\left[I-\beta G_{-S}\right]}^{\text{T}}+{\left[I-\beta G_{-S}\right]}^{-\text{T}}\left[I-\beta G_{-S}\right]\right). \end{array}$$ (33)

Since |*λ* ([**I** − *β***G**_−**S**_]^−1^[**I** − *β***G**_−**S**_]^T^)| = |*λ*([**I** − *β***G**_−**S**_]^−T^[**I** − *β***G**_−**S**_])| = 1 and ([**I** − *β***G**_−**S**_]^−1^[**I** − *β***G**_−**S**_]^T^)^−1^ = ([**I** − *β***G**_−**S**_]^−T^[**I** − *β***G**_−**S**_], let exp(*iw*) and exp(−*iw*) be the eigenvalue of [**I** − *β***G**_−**S**_]^−1^[**I** − *β***G**_−**S**_]^T^ and [**I** − *β***G**_−**S**_]^−T^[**I** − *β***G**_−**S**_], respectively, where *w* ∈ [0, 2π). As a result, it holds that

$$\lambda_{\text{max}}({\left[I-\beta G_{-S}^{}\right]}^{-1}{\left[I-\beta G_{-S}^{}\right]}^{\text{T}}+{\left[I-\beta G_{-S}^{}\right]}^{-\text{T}}[I-\beta G_{-S}^{}])=\text{exp}(iw)+\text{exp}(-iw)=2\text{cos}w\leq 2,$$ (34)

and if and only if *w* = 0, the equality holds.

To summarize, if $X_{-S}\neq X_{-S}^{\text{T}}$ (or $G_{-S}\neq G_{-S}^{\text{T}}$), $1\cdot R_{-S}(G)<1\cdot R_{-S}(\tilde{G})$, and if and only if $X_{-S}=X_{-S}^{\text{T}}$ (or $G_{-S}=G_{-S}^{\text{T}}$), $1\cdot R_{-S}(G)=1\cdot R_{-S}(\tilde{G})$. Property E3 holds.

As Property E2 has proven that liability size is an influencing factor of systemic risk, we here fix the liability size and keep each bank’s total loan and borrowing amounts unchanged in order to precisely study the influence of liability distribution on system risk. Although various liability distributions could exist in real interbank system, Property E3 uncovers that pairwise liabilities symmetrically distributed among the banks, without changing the liability size, can lead to a smaller system risk degree irrespective of the variety of liability distributions.

### 4.4 Risk measurements as a function of bank size

Here we define the bank size as the amount of total liquidity a bank holds. Considering the bank size as the main factor and keeping the lending-borrowing matrix **X** unchanged, when bank *i* does not belong to the initial default set **S** and its size becomes larger, this subsection further checks whether the defined risk degree can reflect the change of bank size. The following Property E4 provides the result.

[**Property E4**] For any given system **G**, a new system $\tilde{G}$ is generated by keeping the lending-borrowing matrix **X** unchanged and the size of bank *i* becomes larger, that is, $X(G)=X(\tilde{G})$ and $al_{i}(G)<al_{i}(\tilde{G})$. Then, for any given default set **S** that does not contain bank *i*, it holds that $r_{i,S}(G)<r_{i,S}(\tilde{G})$ and $rv_{S}(G)>rv_{S}(\tilde{G})$.

**Proof**. Based on the operation described here, the relationship between ${\tilde{G}}_{-S}$ and **G**_−**S**_ is

$$\{\begin{array}{ll} {\tilde{g}}_{ii}=\frac{al_{i}(G)}{al_{i}(\tilde{G})}g_{ii}+1-\frac{al_{i}(G)}{al_{i}(\tilde{G})}, & \text{when}\;j=i,\\ {\tilde{g}}_{ij}=\frac{al_{i}(G)}{al_{i}(\tilde{G})}g_{ij}, & \text{when}\;j\neq i,\\ {\tilde{g}}_{kj}=g_{kj}, & \text{when}\;k\neq i. \end{array}$$ (35)

Then, we have $I-{\tilde{G}}_{-S}=T_{i}\cdot \left(I-G_{-S}\right)$ and, further, $I-\beta {\tilde{G}}_{-S}=\left(I-\beta G_{-S}\right)+\beta (T_{i}-I)\cdot \left(I-G_{-S}\right)$, where **T**_*i*_ is a #(**N** − **S**) × #(**N** − **S**) diagonal matrix whose *i*th element is $al_{i}(G)/al_{i}(\tilde{G})$ and the remaining elements are all 1. Then, $r_{i,S}(\tilde{G})$ can be expressed as a function of **T**_*i*_ as follows, where **1**_*i*_ is an #(**N** − **S**) vector whose *i*th element is 1 and the remaining elements are 0. Then, we further have

$$r_{i,S}(\tilde{G})=\frac{1_{i}\cdot {\left[I-\beta {\tilde{G}}_{-S}^{}\right]}^{-1}\cdot 1_{}^{\text{T}}}{n}=\frac{1_{i}\cdot {\left[\left(I-\beta G_{-S}\right)+\beta (T_{i}-I)\cdot \left(I-G_{-S}\right)\right]}^{-1}\cdot 1_{}^{\text{T}}}{n}.$$ (36)

Here, (**I** − *β***G**_−**S**_) + *β*(**T**_*i*_ − **I**) ⋅ (**I** − **G**_−**S**_) = (**I** − *β***G**_−**S**_)[**I** + *β*(**I** − *β***G**_−**S**_)^−1^(**T**_*i*_ − **I**) ⋅ (**I** − **G**_−**S**_)], and note that

$$\begin{array}{l} \frac{d\left(1_{i}\cdot {\left[\left(I-\beta G_{-S}\right)+\beta (T_{i}-I)\cdot \left(I-G_{-S}\right)\right]}^{-1}\cdot 1^{\text{T}}\right)}{d{\left(T_{i}-I\right)}_{i}}\\ =-\beta 1_{i}\cdot {\left[I-\beta {\tilde{G}}_{-S}\right]}^{-1}diag(1_{i})\left(I-G_{-S}\right){\left[I-\beta {\tilde{G}}_{-S}\right]}^{-1}\cdot 1^{\text{T}}\\ <-\beta 1_{i}\cdot {\left[I-\beta {\tilde{G}}_{-S}\right]}^{-1}diag(1_{i})\left(I-\beta G_{-S}\right){\left[I-\beta {\tilde{G}}_{-S}\right]}^{-1}\cdot 1^{\text{T}}\\ =-\beta 1_{i}\cdot {\left[I-\beta {\tilde{G}}_{-S}\right]}^{-1}\cdot 1^{\text{T}}<0. \end{array}$$ (37)

Thus, **1**_*i*_ ⋅ [(**I** − *β***G**_−**S**_) + *β*(**T**_*i*_ − **I**) ⋅ (**I** − **G**_−**S**_)]^−1^ ⋅ **1**^T^ > **1**_*i*_ ⋅ (**I** − *β***G**_−**S**_)^−1^ ⋅ **1**^T^. That is, $r_{i,S}(\tilde{G})>r_{i,S}(G)$. In addition, by replacing $1_{i}^{\text{T}}$ with **1**^T^ in Eqs (36) and (37), we can immediately obtain that

$$\frac{1\cdot {\left[\left(I-\beta G_{-S}\right)+\beta (T_{i}-I)\cdot \left(I-G_{-S}\right)\right]}^{-1}\cdot 1_{}^{\text{T}}}{n}>\frac{1\cdot {\left(I-\beta G_{-S}\right)}^{-1}\cdot 1_{}^{\text{T}}}{n},$$ (38)

which equals $rv_{S}(G)>rv_{S}(\tilde{G})$.

Property E4 demonstrates the intuition that a larger bank size decreases the systemic risk, which means larger size is a good buffer for insolvency contagion, and indicates that the proposed measurements can well reflect the effect of bank size on system risk.

### 4.5 Risk measurements as a function of the discount factor

This subsection focuses on how each bank’s discount factor *η*_*i*_ affects the defined risk distance and risk degree. Intuitively, an increase in *η*_*i*_ means a stronger ability to liquidate assets; thus, systemic risk should decrease. Here, for any given default set **S**, we check whether the risk distance from bank *i* to **S** increases and the corresponding risk degree *rv*_**S**_ decreases. The following Property E5 provides the answer.

[**Property E5**] For any given default set **S**, if there exists a bank set Ξ such that Ξ ∩ **S** = Φ and ${\tilde{\eta }}_{i}>\eta_{i}$ for any *i* ∈ Ξ in the new system $\tilde{G}$, then the risk distance *r*_*i*,**S**_ is longer in $\tilde{G}$ and the risk degree $rv_{S}(\tilde{G})$ is smaller compared to the original **G**, where *i* ∈ Ξ.

**Proof**. The relationship between $\tilde{G}$ and **G** is $I-{\tilde{G}}_{-S}=Q_{i}\cdot \left(I-G_{-S}\right)$, and further, $I-\beta {\tilde{G}}_{-S}=\left(I-\beta G_{-S}\right)+\beta (Q_{i}-I)\cdot \left(I-G_{-S}\right)$, where **Q**_*i*_ is a #(**N** − **S**) × #(**N** − **S**)diagonal matrix whose *i*th element is $al_{i}(G)/(al_{i}(G)+({\tilde{\eta }}_{i}-\eta_{i})(p_{i}+s_{i}))$ for *i* ∈ Ξ and the remaining elements are all 1. Then, this problem has the same structure with Property E4, and therefore, according to the conclusion provided in Property E4, Property E5 holds.

Property E5 validates the intuition that a rise in the discount factor of some banks will increase the risk distance and decrease the level of system risk. More importantly, the proposed measurements make it possible to reflect the effect of the discount factor, which implies that the effective liquidity management of every bank will benefit the safety of a financial system.

## 5 Conclusions, discussions and future work

This paper belongs to the growing literature that focuses on designing appropriate measurements to evaluate systemic risk in financial networks [28–30]. Specifically, we aim to measure two kinds of risks: one is the susceptibility of each bank to the distress of an initial default set from the microscopic angle, and the other is the the degree of the systemic failures due to contagion of counterparty risk from the macroscopic angle. To this end, this paper proposed a series of computationally tractable measurements that are risk distance, risk degree and *m*-order risk degree, among which the first one captures the first kind of risk and the latter two capture the second kind. Furthermore, this paper also uncovers their basic and extended properties. Regarding the basic properties, we show that the risk distance satisfies non-negativity, asymmetry and monotonicity, and that the risk degree and the m-order risk degree follows monotonicity and heterogeneity. In particular, heterogeneity implies that our measurements support neither “too central to fail” [31] nor “too diverse to fail” [32], owing that different orders reflect different dimensions. On the other hand, the proven extended properties show that our measurements are able to reflect the effect of bank size, liability size, liability distribution and the discount factor on the default risk of one bank and also on the failures of the entire system. Moreover, the rationality of our measurements is embodied not only in the proven basic and extended properties but also in the relationship between the risk distance and financial contagion. From the perspective of methodology, the proposed risk distance is a node-level microscopic indicator that reflects the default likelihood of each remaining bank when an initial default set is given., while the proposed risk degree and *m*-order risk degree is a system-level macroscopic indicator that reflects the collapsing force of the given default sets. Both of them inherit the basic framework of the classical Katz-Bonacich centrality and establish a Markov transfer matrix with absorbing states.

Based on the proposed measurements, this paper also provides some implications for guiding how to decrease or prevent the systemic risk of interbank systems: (1) since liability size influences systemic risk, it is an effective method for controlling the level of total credits within normal levels, such as deleveraging; (2) a symmetric liability distribution between pairwise banks will create a safer system under the precondition that the borrowing amount of each bank is equal to its loan amount; (3) the targeted liquidity injection is useful because banks with large bank size or liquidities are good buffers in the way of insolvency contagion; (4) enhancing the ability of banks to liquidate their assets or raise their capital adequacy ratios will decrease systemic risk.

Two issues are further discussed here: one is related to the problem of missing information and the other is related to varieties of measures of controlling the system risk. With regards to the first issue, one precondition of this paper is to know all the information about each bank’s balance sheet. However, granular data on financial networks is often lacking and the limitedness of the information available always exists in real practice. Thus, how to achieve the missing information and how to make decisions based on partial information also become two potential problem, although they are not deeply discussed in our work. Fortunately, [33] and [34] provided some feasible approaches to cope with the problem. Based on their work, once the missing information is estimated, our approach and the main results can also be adopted to analyze the risk contagion of interbank system. With regards to the second issue, apart from the above suggested measures of controlling the system risk, many others are also potentially useful in the context of network-based interbank system. For example, [35] uncovered how the topological features of network structures influence the risk contagion and suggested avoiding the measures such as market integration and diversification to decrease system stability, and [36] studied how types of debt contracts affected the system risk and suggested a more suitable type of contract that guaranteed for a unique Pareto efficient clearing payment vector. Accordingly, the mentioned two papers enrich the measures to reduce the system risk, which guides our future work to extend our proven properties.

In our opinion, it is meaningful to explore the relationship between the network structure and the proposed *m*-order system value because doing so may provide specific guidance on how to design a robust network structure under certain conditions. To this end, we suggest plotting all the different-order risk degrees on one line graph for finding out a sudden change on risk degrees in the process of adding banks into the given initial default set, which is a visual way to identify the influence of financial contagion. Moreover, note that the fix-point method captures the system risk under another mechanism of risk contagion different with ours [1], and therefore it will be interesting to check whether the fix-point method also shares the similar properties with ours. In addition, since this paper focuses on only interbank interactions with a balance-sheet mechanism, it would be interesting to study a more general framework that extends the interbank system to a generalized financial system that includes more types of financial institutions and interaction.
